# KAT5: the epigenetic regulator of central nervous system diseases

**DOI:** 10.3389/fnmol.2026.1887674

**Published:** 2026-09-03

**Authors:** Xiaoming Xin, Lingjuan Wu, Meichen Liu, Tian Liu, Zihan Yuan, Liang Zhang, Tongqing Duan, Lei Zhang, Xianmin Zhu

**Affiliations:** 1 Shanghai University of Medicine and Health Sciences, Shanghai, China; 2 Shanghai University of Traditional Chinese Medicine, Shanghai, China; 3 Qingdao Eighth People’s Hospital, Qingdao, China; 4 Qingdao Cardiovascular Disease Hospital, Qingdao, China; 5 Jinshan Central Hospital Affiliated to Shanghai University of Medicine and Health Sciences, Shanghai, China; 6 Shanghai Academy of Sciences and Technology Institute of Model Animals Transformation, Shanghai, China

**Keywords:** acetylation, CNS diseases, epigenetic modification, histones, KAT5

## Abstract

Abnormal epigenetic modifications are involved in central nervous system (CNS) diseases. Histones play a crucial role in chromatin structure and function, whose post-translational modifications significantly impact gene expression and chromatin dynamics. Histone acetylation, governed by the balance between histone acetyltransferases (HATs) and histone deacetylases (HDACs), is one of the key modulators of chromatin accessibility and transcriptional activity. Lysine acetyltransferase 5 (KAT5, aka TIP60), a member of the MYST subfamily of HATs, is involved in many cellular processes, including DNA repair, apoptosis, and cell cycle control. Notably, the dysfunction of KAT5 has been implicated in several CNS diseases. In this review, we explored the roles of KAT5 in CNS pathophysiology, emphasizing its involvement in neurological disorders and its potential as a therapeutic target. This review sheds light on the epigenetic mechanisms in CNS diseases mediated by KAT5 and provides valuable information for potential treatment strategies.

## 1 Introduction

Central nervous system (CNS) diseases are increasingly recognized to involve dysregulation of epigenetic modifications (Wey et al., 2016). The term “epigenetics” was first introduced by Conrad Waddington to describe heritable changes in cell phenotypes that are not related to alterations in the DNA sequence (Dawson and Kouzarides, 2012). Histone modifications are one of the epigenetic mechanisms. Histones are proteins that form the octameric core of eukaryotic nucleosomes, consisting of two copies of each histone H2A, H2B, H3, and H4. DNA wraps around this histone core to form nucleosome, the basic unit of chromatin (Mai et al., 2025). Post-translational modifications of histones have significant impact on chromatin structure, including methylation, acetylation, phosphorylation, ubiquitinylation, etc. Histone acetylation occurs at the ε amino groups of evolutionarily conserved lysine residues located at the N-termini. The stable acetylation levels of core histones result from the balance between the activities of histone acetyltransferases (HATs) and histone deacetylases (Heard and Martienssen, 2014; HDACs) (de Ruijter et al., 2003). HATs catalyze the transfer of an acyl moiety from acyl coenzyme A to lysine residues on the N-terminal tails of the histones. HDACs function to catalyze removal of acetyl groups from specific and conserved histone lysine residues, causing a compacted and transcriptionally repressive chromatin environment (Mai et al., 2025). However, subsequent studies revealed that the regulatory functions of HATs and HDACs are not restricted to histones; these enzymes can also modify a wide range of non-histone proteins. Therefore, some researchers have proposed that HATs and HDACs should be renamed as lysine acetyltransferases (KATs) and lysine deacetylases (KDACs), respectively, to more accurately reflect their broader substrate specificity and biological functions.

Based on sequence similarity, substrate preference, and functional characteristics, KATs can be classified into several distinct families (Mihaylova and Shaw, 2013). Lysine acetyltransferase 5 (Kat5) is one of the most representative proteins in the MYST subfamily of HATs (Mai et al., 2025). The gene encoding KAT5 is located at 11q13.1 and consists of 14 exons. It plays a role in various cellular processes, including cellular signaling, DNA damage repair, cell cycle and checkpoint control, and apoptosis (Sapountzi et al., 2006). Previous studies have shown that the HAT activity of KAT5 plays a crucial role in controlling the neuronal-specific gene expression profile and the function of the central nervous system (CNS) (Lorbeck et al., 2011). Therefore, it is essential to discuss the role of KAT5 in CNS diseases.

**SCHEME 1:**
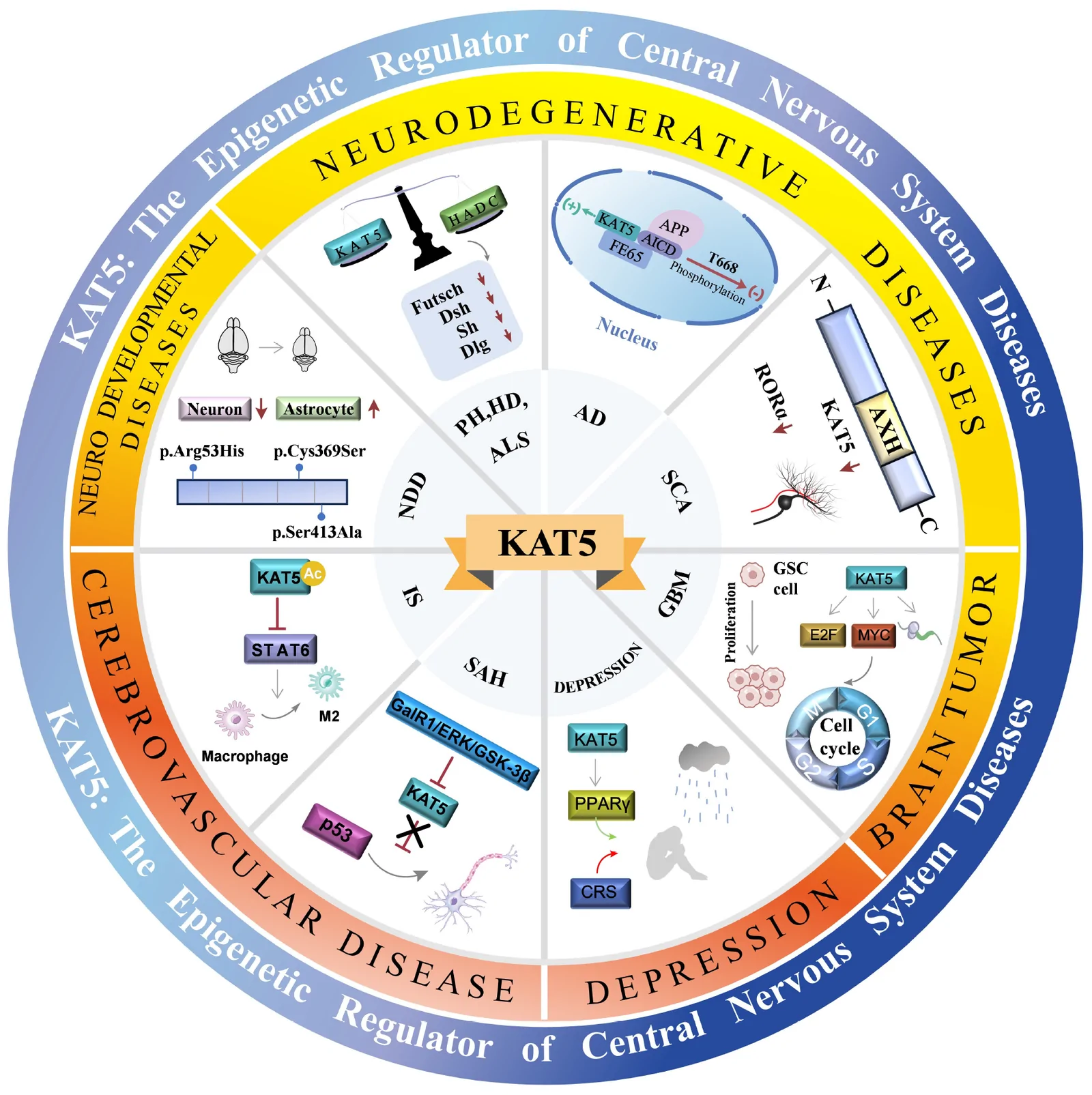
The significant role of KAT5 in neurological diseases.

In this review, we focused on a detailed examination of KAT5 as an epigenetic regulator within the CNS. We summarized its pathological involvement in neurological diseases (Table 1). A better understanding of KAT5 and its diverse roles in CNS diseases will provide valuable insights for developing therapies targeting histone acetylation in the treatment of CNS disorders (Scheme 1).

**TABLE 1:** KAT5 expression and functional mechanisms in central nervous system diseases.

Disease		Model	KAT5 expression (experimental model)	Key molecular mechanism	Functional/phenotypic outcome	References
Neurodegenerative diseases	AD	Postmortem AD hippocampal tissues in humans and Drosophila mushroom body	↓	APP (AICD)-FE65-KAT5; KAT5→AICD/AFT-Notch (NICD); KAT5→BEST1 and GADD45G	Transcriptional dysregulation and cognitive impairment	(Bhatnagar et al., 2025; Kolbe et al., 2016; Li and Rasmussen, 2020; Mai et al., 2025)
	PD	Drosophila (α-synuclein A30P)	↓	Tip60/HDAC2→futsch, dsh, sh, dlg↓(H4K12ac and H4K16ac)	Abnormal synaptic morphology, motor disorders, and short-term memory deficits	(Beaver et al., 2020)
	HD	Drosophila (Htt128Q)	↑			
	ALS	Drosophila (Vap-33-1)	↓			
	SCA	Mouse [ATXN1(82Q)]	↑	KAT5-ATXN1 (amino acids 425–547)→RORα	Molecular layer (postnatal week 2↓↓, postnatal week 16↓)	(Tejwani and Lim, 2020; Serra et al., 2006)
		[ATXN1(82Q) × Tip60^ + ⁣/−^]	↑	KAT5-ATXN1 (AXH, a phospho-mimicking Asp at 776) → RORα	Molecular layer↓(Tip60+/2 was no longer protective at 20 weeks), rotarod deficit and Purkinje cell atrophy	(Gehrking et al., 2011)
Brain tumor	GBM	NSG Mouse (GSCs, LECs)	↑	CCL21-CCR7→KAT5-HMGCS1→cholesterol synthesis→tumor growth	Accelerated tumor growth	(Zhao et al., 2024)
		NSG mouse (GSCs)	↑	KAT5 knockdown→E2F/MYC↓, G0-like states, invasiveness↓	80% of cells in three cycling cell clusters, tumor volume↑, survival probability↓	(Mihalas et al., 2025)
**Disease**		**Model**	**KAT5 expression**	**Key molecular mechanism**	**Functional/phenotypic outcome**	**References**
Depression		C57B/6J mouse (CRS)	↓	CRS→KAT5↓→depression, over-expression KAT5→ PPARγ↑	Immobility time↑ short-term spatial memory↓ Latency↓ frequency and permanence time to (in) the darkroom↓	(Ji et al., 2022)
Cerebrovascular disease	SAH	SD rat (endovascular perforation)	↑	GalR1↑→ERK↑→ GSK-3β↑→ TIP60↓; TUNEL↓;Bax↓, Bcl-2↑	Neuronal apoptosis and degeneration; modified Garcia score↓	(Shi et al., 2021)
	IS	SD rat (tMCAO)	↑	KAT5 knockdown→STAT6 acetylation ↓, phosphorylation ↑ M1→M2, IL-6↓, IL-10↑	Infarct volume↑; disorganized neurons and disordered cytoplasm, and neurofibrils ↓	(Li et al., 2025)
Neurodevelopmental disorders		K562 cells in human (p.Arg53His, p.Cys369Ser, p.Ser413Ala)	Heterozygous *de novo* missense variant	Histone H4 tail acetylation ↓ HDAC4↑ LHX9 and KIRREL3↓ PER1↑	Sleep disturbance, cerebellar atrophy, and facial dysmorphisms	(Humbert et al., 2020)
		Tip60^F/F^; Nes-Cre+ mouse	↓	Scrt1↓ Gcg↑	Microcephaly, poor neurosphere formation, proliferation defects, neural differentiation defects, and accelerated astrocytic differentiation	(Tominaga et al., 2023)

## 2 Major functions and basic construct of KAT5

KAT5 is a multi-domain protein, comprising an N-terminal chromodomain, a C-terminal MYST acetyltransferase domain, and a C-terminal nuclear receptor interaction box (NR-box). Among these, the MYST domain constitutes the core functional region of TIP60, containing two key sub-structures: the conserved HAT domain, which binds acetyl-CoA and substrates, and the Cys-Cys-His-Cys zinc finger, which is critically essential for acetyltransferase activity (Sapountzi et al., 2006). The major protein partners of KAT5 can be generally categorized into two classes. The first class comprises substrate proteins that are directly acetylated by KAT5, including PC4 (positive coactivator 4) (Shi et al., 2024), cGAS (cyclic GMP-AMP synthase) (Song et al., 2020), and histones (e.g., H4 and H2AX). The second class consists of proteins that either form functional complexes with KAT5 or engage in direct physical interactions with it, including PARP1 [poly(ADP-ribose) polymerase 1], the NUA4 complex, transcription factors (e.g., c-Myc and E2F) (Mihalas et al., 2025), and KCC3 (potassium-chloride cotransporter 3) (Hishikawa et al., 2021).

## 3 KAT5 in disorders of the CNS

### 3.1 Neurodegenerative diseases

Neurodegenerative diseases (NDs) are a heterogeneous group of disorders characterized by the progressive death of neurons in the brain and/or spinal cord. NDs differ in the molecular pathways underlying the disease and subsequent clinical symptoms, depending on the particular subset of neurons and/or the areas of the nervous system most severely damaged. Despite the broad molecular and phenotypic diversity exhibited by NDs, they share some common features, such as mitochondrial dysfunction, dysregulation of similar molecular/cellular pathways, protein misfolding and aggregation, and the loss of chromatin dynamics (Przedborski et al., 2003).

#### 3.1.1 Alzheimer’s disease (AD)

Alzheimer’s disease (AD) refers to the onset and course of cognitive and functional decline associated with age together with a particular neuropathology (Soria Lopez et al., 2019). In human postmortem AD hippocampal tissues and an AD-associated amyloid precursor protein (APP) neurodegenerative *Drosophila* model, KAT5 protein levels were reduced, leading to decreased KAT5-mediated acetylation at histone marks and concomitant repression of key neuroplasticity genes. Elevating KAT5 levels can ameliorate this condition. Furthermore, it is sufficient to prevent multiple neural processes impaired in the early stages of AD, including axonal outgrowth/transport, learning/memory, circadian rhythm, synaptic plasticity, locomotor function, and apoptosis-driven neurodegeneration. Collectively, these studies indicate that KAT5 plays an overall neuroprotective role across multiple neural cognitive circuits impaired in AD (Bhatnagar et al., 2025).

KAT5 regulates AD through multiple mechanisms. Familial AD is associated with mutations in the APP gene. If APP is cleaved, the APP intracellular domain (AICD) formed interacts with FE65 and KAT5 to form the AFT complex. The abnormal formation and recruitment of the AFT complex may lead to the onset of AD by disrupting neuronal target genes. KAT5 can also cross-regulate the balance between AICD/AFT and Notch intracellular domain (NICD) signaling; Any imbalance in this process can cause disruptions in neuronal gene expression programs. In conclusion, the downregulation of KAT5 disrupts the HAT/HDAC balance, leading to transcriptional dysregulation, which is a key early step in the pathogenesis of AD (Li and Rasmussen, 2020; Mai et al., 2025). Furthermore, in an AD *Drosophila* model, Kat5 overexpression promotes neuronal survival by activating pro-survival genes and repressing pro-apoptotic factors. It also restores axonal growth and transportation in circadian neurons which are impaired by APP overexpression, and rescues the related behavioral phenotypes such as sleep and locomotion. KAT5 aggregates in the AD brain and participates in the nuclear sphere formation through interaction with APP and FE65. The nuclear sphere is related to DNA repair, gene expression and cell cycle abnormalities (Kolbe et al., 2016). It can be negatively regulated by APP T668 phosphorylation but enhanced by KAT5. KAT5 works synergistically with FE65 to regulate nuclear sphere-dependent gene expression and may participate in the pathological process of AD by affecting genes such as BEST1 and GADD45G (Loosse et al., 2016). Taken together, KAT5 is a key HAT in the DNA damage response, regulating chromatin relaxation and repair gene expression. As DNA damage is considered an early event in AD, KAT5 may participate in neuronal death or survival decisions through epigenetic mechanisms. KAT5 exerts neuroprotective effects in AD by regulating AICD-mediated gene expression, DNA damage response, and microtubule acetylation. Enhancing KAT5 activity may become a novel epigenetic therapeutic strategy.

Xu et al. (2014) found Kat5 is constitutively expressed in the adult Drosophila mushroom body (MB). Loss of Kat5 activity leads to abnormal MB axonal development and impaired immediate recall memory, while overexpression results in no morphological abnormalities. Chromatin immunoprecipitation sequencing (ChIP-seq) revealed that Kat5 targeted genes are enriched in functional pathways related to cognition, and these key genes are abnormally expressed in mutants. Importantly, APP-induced learning and memory deficits can be significantly ameliorated by increasing Kat5 levels in the MB. This study reveals that KAT5 influences cognitive function through epigenetic transcriptional regulation and highlights the potential of targeted HAT activators for treating cognitive impairment associated with neurodegenerative diseases (Xu et al., 2014).

Another model of preclinical mild cognitive impairment (MCI) in AD revealed that disruption of neuronal acetylation homeostasis in the brain by loss of KAT5 HAT activity and increased HDAC2 activity can alter KAT5 HAT/HDAC2 balance which leads to inappropriate recruitment of HDAC2 to neuroplasticity genes, resulting in their repression (Bhatnagar et al., 2025; Panikker et al., 2018). Therefore, increasing KAT5 in AD brains restores HAT/HDAC2 balance, which maintains neuroplasticity gene expression, brain morphology, and cognition.

#### 3.1.2 Parkinson’s disease (PD), Huntington’s disease (HD), and amyotrophic lateral sclerosis (ALS)

Previous studies have shown that histone acetylation dysregulation contributes to age-related cognitive impairment and age-associated NDs, including Parkinson’s disease (PD), Huntington’s Disease (HD) and Amyotrophic Lateral Sclerosis (ALS). However, studies examining the relationship between HATs and these three NDs remain limited. PD is characterized by the degeneration and loss of dopaminergic neurons, primarily in the substantia nigra pars compacta within the ventral midbrain. HD is a fatal, progressive neurodegenerative disorder caused by an expanded CAG repeat in the huntingtin gene. ALS is referred to as a fatal ND disease caused by the degeneration of motor neurons in the cerebral cortex, brainstem, and spinal cord, resulting in generalized weakness, muscle atrophy, and paralysis. Beaver et al. (2020) first observed in the early stages of various Drosophila neurodegenerative disease models (i.e., PD, HD, and ALS) that the balance between Kat5 HAT and HDAC2 was disrupted, accompanied by the epigenetic repression of common Kat5 target genes involved in synaptic plasticity, a phenomenon reminiscent of that seen in AD. Genes such as Shaker (Sh), Futsch, Disc large (Dlg), and Dishevelled (Dsh) were directly regulated by Kat5, and their expression was suppressed in the early stages of APP-mediated neurodegenerative pathology. This suppression could be reversed by increasing Kat5 levels in the brain. Notably, Futsch and Dsh were downregulated in all three ND models, while Sh and Dlg showed specific downregulation in HD and PD models, respectively. Additionally, pronounced morphological changes were observed in ALS and PD models. Though no significant changes were noted in the HD model. Still, functional abnormalities may exist. Moreover, increasing the levels of Kat5 in the MB region of the Drosophila larval brain was able to restore early motor defects in all three NDs, but it only partially recovered short-term memory deficits in the PD model, with limited effects on the HD and ALS models (Beaver et al., 2020; Mai et al., 2025).

#### 3.1.3 Spinocerebellar ataxia (SCA)

Ataxia represents a disordered clinical syndrome and is also used to refer to a group of specific neurodegenerative diseases. Spinal cerebellar ataxia (SCA) is one of the genetically heterogeneous autosomal dominant progressive disorders. Its clinical features include loss of balance and coordination, accompanied by speech impairment. SCA is heterogeneous and currently tends to be classified based on the chronological order of the discovery of the pathogenic gene or locus. Each subtype is named SCAn (Gehrking et al., 2011; Klockgether et al., 2019; Matilla-Dueñas et al., 2014).

One of the key domains of the ataxin-1 protein, the ataxin-1/HBP-1 (AXH) domain, mediates its normal function and pathogenic behavior in SCA type 1. In Purkinje cells of SCA1 transgenic mouse models, Kat5 was found to directly bind to the AXH domain of the mutant ATXN1, resulting in the loss of normal transcriptional activation function of Kat5 as a co-activator, decreased expression of retinoic acid-related receptor alpha (RORα), and dysfunction of Purkinje cells. A partial reduction of Kat5 can delay the progression of the disease at certain stages, but the long-term effect may disappear or even reverse (Gehrking et al., 2011; Serra et al., 2006; Tejwani and Lim, 2020).

### 3.2 Brain tumor

KAT5 plays multiple roles in cancer, influencing key functions such as DNA repair and transcriptional regulation through direct or indirect protein interactions. However, the role of KAT5 varies depending on the cancer type, where it can act either as a tumor suppressor or as an oncogene (Judes et al., 2015). KAT5 enhances the transcriptional activity of NF-κB (Nesbit et al., 1999), stabilizes c-Myc protein (Gerritsen et al., 1997), and promotes the progression of various cancers. KAT5 acetylates and activates the androgen receptor (AR), facilitating the onset of prostate cancer (Coffey et al., 2012). In colorectal cancer cells, knockdown of BRD8, a functional auxiliary subunit of the p400/Tip60 complex, leads to reduced H4K16 acetylation activity of KAT5/TIP60, resulting in DNA repair defects and p53-dependent apoptosis (Lashgari et al., 2018). On the other hand, KAT5 recruits histone deacetylases to inhibit the transcriptional activity of c-Myb and downregulates STAT3 expression (Judes et al., 2015). Additionally, KAT5 acetylates the K120 site of p53, inducing apoptosis (Xu et al., 2009). KAT5 also acetylates and regulates the balance between BRCA1 and 53BP1, influencing the development of breast cancer (Tarsounas and Sung, 2020).

In brain cancer, glioblastoma (GBM) is the most aggressive form (Tirpe et al., 2023). Histopathological studies reveal that certain cells within the GBM tumor are in a transient or prolonged quiescent state (G0-like states). The cell cycle may be linked to the cellular diversity of GBM and its resistance to chemoradiotherapy. KAT5 has been identified as a key regulatory factor in the transition to G0-like states in GBM. KAT5 activity inhibits the emergence of quiescent subpopulations, which possess characteristics of neurodevelopmental progenitor cells. Furthermore, KAT5, in conjunction with protein translation, regulates the E2F and MYC transcriptional networks, promoting the self-renewal of GBM stem-like cells (GSCs). In contrast, inhibition of KAT5 causes GSCs and GSC-derived tumor cells to transition into G0-like states, characterized by neurodevelopmental features. In animal models, reduced KAT5 activity significantly suppresses tumor growth, decreases cell proliferation, and exhibits a synergistic effect when combined with standard therapies (temozolomide ++ radiotherapy), significantly extending the survival of mice. Analysis of primary glioma samples shows a significant positive correlation between KAT5 activity and protein translation rate in tumor cells. High-grade gliomas exhibit significantly higher KAT5 activity and protein translation rates compared to low-grade gliomas (Mihalas et al., 2025).

In addition, KAT5 may exert its oncogenic function through the regulation of cholesterol metabolism. Zhao et al. (2024) demonstrated that lymphatic endothelial-like cells (LECs) identified in the GBM parenchyma secrete CCL21, which triggers widespread acetylation changes in GSCs. Among these modifications, acetylation at lysine 273 (K273) of HMGCS1, a key enzyme in the cholesterol biosynthesis pathway, was upregulated. By knocking out currently known acetyltransferases in GSCs, they showed that KAT5 deletion most effectively reduced HMGCS1 K273 acetylation, thereby suppressing GSC proliferation (Zhao et al., 2024).

### 3.3 Depression

Depression is one of the most prevalent, diverse, and complex psychiatric syndromes worldwide. The definition of the depressive syndrome includes symptoms of sadness or depressed mood and/or diminished interest in daily activities, accompanied by other psychological symptoms, including difficulty concentrating, feelings of hopelessness or expressed guilt, and thoughts of death or suicide. Additionally, Patients may experience somatic symptoms such as fatigue, changes in sleep and appetite, psychomotor retardation, or agitation (Simon et al., 2024). Epigenetics plays a crucial role in the pathophysiology of depression. For example, in major depressive disorder, chromatin condensation leads to reduced accessibility of transcription factors to their binding sites, which may impair the effectiveness of antidepressants. In such cases, the combined use of antidepressants and HDAC inhibitors may help treat patients with treatment-resistant major depression (Fuchikami et al., 2016; Penney and Tsai, 2014).

The chronic restraint stress model can induce cognitive impairments and depressive-like behaviors. Wang et al. (2022) injected KAT5 overexpression lentiviral vector into the hippocampus of mice before the chronic restraint stress (CRS) experiment and found that KAT5 could ameliorate CRS-induced damage to long-term potentiation, dendritic spine density loss, and the decrease in synaptic proteins. Additionally, KAT5 was validated as a specific coactivator of PPARγ. Peroxisome proliferator-activated receptor (PPAR), a regulator of energy metabolism, is considered a potential therapeutic target for depression (Wang et al., 2022). They discovered that PPARγ antagonists significantly weakened the protective effect of KAT5 overexpression in CRS mice, indicating that the antidepressant efficacy of KAT5 is dependent on PPARγ activity (Ji et al., 2022).

### 3.4 Cerebrovascular disease

Cerebrovascular disease (CeVD) is defined as a range of diseases that lead to cerebral ischemia or hemorrhage. This category includes stroke, carotid vertebral or intracranial stenosis, aneurysms, and vascular malformations (Osorio et al., 2022). Stroke is commonly classified into ischemic stroke(IS) and hemorrhagic stroke. Subarachnoid hemorrhage (SAH), a subtype of hemorrhagic stroke, refers to a series of syndromes caused by the rupture of intracranial aneurysms, leading to the influx of blood into the subarachnoid space (Li and Chen, 2023). Shi et al. (2021) found that 24 h after SAH, the levels of phosphorylated KAT5 were significantly elevated. Through the GalR1/ERK/GSK-3β pathway, the activity of KAT5 was inhibited, thereby blocking the p53-mediated neuronal apoptosis program (Shi et al., 2021). Neuroinflammation leads to secondary brain injury. Microglial cells in IS, which exhibit pro-inflammatory (M1) and anti-inflammatory (M2) phenotypes during this process, play a critical role in neuroinflammation. Li et al. (2025) discovered that during early IS, KAT5 can acetylate and inhibit the activity of the key transcription factor signal transducer and activator of transcription 6 (STAT6), thereby promoting M2 polarization (Li et al., 2025).

### 3.5 Neurodevelopmental disorders

Neurodevelopmental disorders (NDDs) encompass a range of conditions that affect brain function and development, including autism, attention deficit hyperactivity disorder, dyslexia, and other cognitive impairments, affecting millions of children worldwide (Grandjean and Landrigan, 2014; Yiliyaer et al., 2025). Brain development is strictly regulated by epigenetic factors, and variations in genes associated with lysine acetyltransferases, such as KAT5, KAT6A, and KAT6B (Mousavi and Yang, 2025; You et al., 2015) have been found in individuals with NDDs characterized by intellectual disabilities and malformations. Humbert et al. (2020) reported three cases of neurodevelopmental delay, cerebellar atrophy, epilepsy, and intellectual disability caused by dominant missense mutations in the KAT5 gene. These missense mutations severely impair the histone acetyltransferase activity of the KAT5 protein, particularly its ability to acetylate histone H4. Furthermore, they observed downregulation of neurodevelopmental genes such as LHX9 and KIRREL3 in patient fibroblasts (Humbert et al., 2020).

Tominaga et al. (2023) established a neural stem cell-specific Kat5^*F*/*F*^; Nes-Cre^+^ (Kat5 cKO) mice model. Postnatally, Kat5 cKO mice exhibited brachycephaly and microcephaly, with significant cortical thinning. They further discovered a substantial reduction in proliferating cells in the embryonic brains of KAT5 cKO mice, with cell cycle arrest occurring in the M phase. Additionally, neural stem/progenitor cells prematurely switched from generating neurons to producing astrocytes, and the distribution of neuronal migration markers was severely disrupted (Tominaga et al., 2023).

## 4 Limitations and future perspectives

Although KAT5 has gradually emerged as a promising therapeutic target in central nervous system (CNS) disorders and other diseases, its clinical translation still faces several critical challenges. KAT5 is broadly expressed across various human tissues and cell types, with relatively high expression levels in the brain, suggesting its important role in neurological functions. KAT5 regulates the transcription of genes essential for cognitive function and neuronal survival, particularly in the hippocampal CA1 region, thereby contributing to the maintenance of neuronal homeostasis.

However, due to the complexity and diversity of KAT5 biological functions, translating KAT5-targeted therapeutic strategies into clinical applications remains challenging. First, KAT5 interacts with multiple substrates, and it has been reported to associate with at least 20 interacting proteins. These diverse molecular interactions may determine its substrate specificity, regulatory mechanisms, and cellular functions, leading to distinct biological outcomes depending on the cellular and physiological context. Second, given the broad involvement of KAT5 in transcriptional regulatory networks, global modulation of chromatin acetylation may induce unintended epigenetic alterations and potential toxicity. Therefore, the development of selective KAT5 activators or inhibitors remains technically challenging, and a universal therapeutic strategy may not be applicable across all disease contexts. Future therapeutic approaches should focus on precise regulation of KAT5 based on disease type, tissue origin, and cellular state to achieve more effective and safer clinical applications.

Furthermore, the MYST acetyltransferase domain of KAT5 is highly conserved between Drosophila and humans, exhibiting approximately 89% amino acid sequence similarity and a root-mean-square deviation (RMSD) of < 1 Å between their three-dimensional structural models. This high degree of conservation provides an important foundation for using model organisms to investigate KAT5 functions. However, future studies should increasingly incorporate mammalian models to further evaluate the therapeutic efficacy, pharmacological properties, and translational potential of KAT5-targeting interventions.

Additionally, the histone acetyltransferase activity of KAT5 is jointly regulated by acetylation and ubiquitination. Acetylation enhances its activity, while ubiquitination reduces its activity and promotes its degradation. As both modifications target lysine residues, the interaction between acetylation and ubiquitination warrants further investigation. CNS diseases encompass a wide variety, with complex etiologies. In addition to the diseases mentioned in this paper, numerous studies have demonstrated that abnormal expression of HDACs is associated with CNS disorders such as schizophrenia (Sharma et al., 2008), Rett syndrome (Castro et al., 2013), substance abuse (Ajonijebu et al., 2017), and posttraumatic stress disorder (PTSD) (Bahari-Javan et al., 2012). These findings collectively highlight the critical role of histone acetylation homeostasis in maintaining CNS function. Therefore, precise regulation of KAT5, a key HAT, may offer new combination therapeutic strategies for treating these complex neurological disorders in the future.

## 5 Conclusion

KAT5 emerges as a critical epigenetic regulator in the CNS, with its involvement in the pathogenesis of various neurodegenerative diseases becoming increasingly evident. It regulates the transcription of genes essential for cognitive functions and neuronal viability, particularly in the hippocampal CA1 region. The pathological roles of KAT5 in diseases such as AD and depression highlight its potential as a therapeutic target for restoring normal epigenetic regulation. Future research should focus on unraveling the precise molecular mechanisms by which KAT5 modulates chromatin dynamics in the nervous system and developing targeted therapies that could mitigate or reverse the effects of its dysfunction. Better understanding of KAT5’s intricate involvement in the epigenetic regulation of CNS diseases will pave the way for innovative and precise approaches to treat these debilitating conditions.

## References

[B1] Ajonijebu D. Abboussi O. Russell V. Mabandla M. Daniels W. (2017). Epigenetics: A link between addiction and social environment. *Cell. Mol. Life Sci.* 74 2735–2747. 10.1007/s00018-017-2493-1 28255755 PMC11107568

[B2] Bahari-Javan S. Maddalena A. Kerimoglu C. Wittnam J. Held T. Bähr M. et al. (2012). HDAC1 regulates fear extinction in mice. *J. Neurosci.* 32 5062–5073. 10.1523/JNEUROSCI.0079-12.2012 22496552 PMC6622110

[B3] Beaver M. Bhatnagar A. Panikker P. Zhang H. Snook R. Parmar V. et al. (2020). Disruption of Tip60 HAT mediated neural histone acetylation homeostasis is an early common event in neurodegenerative diseases. *Sci. Rep.* 10:18265. 10.1038/s41598-020-75035-3 33106538 PMC7588445

[B4] Bhatnagar A. Thomas C. Nge G. Zaya A. Dasari R. Chongtham N. et al. (2025). Tip60 HAT activators as therapeutic modulators for Alzheimer’s disease. *Nat. Commun.* 16:3347. 10.1038/s41467-025-58496-w 40199891 PMC11978860

[B5] Castro J. Mellios N. Sur M. (2013). Mechanisms and therapeutic challenges in autism spectrum disorders: Insights from Rett syndrome. *Curr. Opin. Neurol.* 26 154–159. 10.1097/WCO.0b013e32835f19a7 23449173

[B6] Coffey K. Blackburn T. Cook S. Golding B. Griffin R. Hardcastle I. et al. (2012). Characterisation of a Tip60 specific inhibitor, NU9056, in prostate cancer. *PLoS One* 7:e45539. 10.1371/journal.pone.0045539 23056207 PMC3466219

[B7] Dawson M. Kouzarides T. (2012). Cancer epigenetics: From mechanism to therapy. *Cell* 150 12–27. 10.1016/j.cell.2012.06.013 22770212

[B8] de Ruijter A. van Gennip A. Caron H. Kemp S. van Kuilenburg A. (2003). Histone deacetylases (HDACs): Characterization of the classical HDAC family. *Biochem. J.* 370 737–749. 10.1042/BJ20021321 12429021 PMC1223209

[B9] Fuchikami M. Yamamoto S. Morinobu S. Okada S. Yamawaki Y. Yamawaki S. (2016). The potential use of histone deacetylase inhibitors in the treatment of depression. *Prog Neuropsychopharmacol. Biol. Psychiatry* 64 320–324. 10.1016/j.pnpbp.2015.03.010 25818247

[B10] Gehrking K. Andresen J. Duvick L. Lough J. Zoghbi H. Orr H. (2011). Partial loss of Tip60 slows mid-stage neurodegeneration in a spinocerebellar ataxia type 1 (SCA1) mouse model. *Hum. Mol. Genet.* 20 2204–2212. 10.1093/hmg/ddr108 21427130 PMC3090197

[B11] Gerritsen M. Williams A. Neish A. Moore S. Shi Y. Collins T. (1997). CREB-binding protein/p300 are transcriptional coactivators of p65. *Proc. Natl. Acad. Sci. U S A.* 94 2927–2932. 10.1073/pnas.94.7.2927 9096323 PMC20299

[B12] Grandjean P. Landrigan P. (2014). Neurobehavioural effects of developmental toxicity. *Lancet Neurol*. 13 330–338. 10.1016/S1474-4422(13)70278-3 24556010 PMC4418502

[B13] Heard E. Martienssen R. (2014). Transgenerational epigenetic inheritance: Myths and mechanisms. *Cell* 157 95–109. 10.1016/j.cell.2014.02.045 24679529 PMC4020004

[B14] Hishikawa A. Hayashi K. Kubo A. Miyashita K. Hashiguchi A. Kinouchi K. et al. (2021). DNA repair factor KAT5 prevents ischemic acute kidney injury through glomerular filtration regulation. *iScience* 24:103436. 10.1016/j.isci.2021.103436 34877495 PMC8633972

[B15] Humbert J. Salian S. Makrythanasis P. Lemire G. Rousseau J. Ehresmann S. et al. (2020). De novo KAT5 variants cause a syndrome with recognizable facial dysmorphisms, cerebellar atrophy, sleep disturbance, and epilepsy. *Am. J. Hum. Genet.* 107 564–574. 10.1016/j.ajhg.2020.08.002 32822602 PMC7477011

[B16] Ji J. Li S. Jiang Z. Yu J. Sun Y. Cai Z. et al. (2022). Activating PPARβ/δ protects against endoplasmic reticulum stress-induced astrocytic apoptosis via UCP2-dependent mitophagy in depressive model. *Int. J. Mol. Sci.* 23:10822. 10.3390/ijms231810822 36142731 PMC9500741

[B17] Judes G. Rifaï K. Ngollo M. Daures M. Bignon Y. Penault-Llorca F. et al. (2015). A bivalent role of TIP60 histone acetyl transferase in human cancer. *Epigenomics* 7 1351–1363. 10.2217/epi.15.76 26638912

[B18] Klockgether T. Mariotti C. Paulson H. (2019). Spinocerebellar ataxia. *Nat. Rev. Dis. Primers* 5:24. 10.1038/s41572-019-0074-3 30975995

[B19] Kolbe K. Bukhari H. Loosse C. Leonhardt G. Glotzbach A. Pawlas M. et al. (2016). Extensive nuclear sphere generation in the human Alzheimer’s brain. *Neurobiol. Aging* 48 103–113. 10.1016/j.neurobiolaging.2016.08.016 27644079

[B20] Lashgari A. Fauteux M. Maréchal A. Gaudreau L. (2018). Cellular depletion of BRD8 causes p53-dependent apoptosis and induces a DNA damage response in non-stressed cells. *Sci. Rep.* 8:14089. 10.1038/s41598-018-32323-3 30237520 PMC6147888

[B21] Li X. Chen G. (2023). CNS-peripheral immune interactions in hemorrhagic stroke. *J. Cereb. Blood Flow Metab.* 43 185–197. 10.1177/0271678X221145089 36476130 PMC9903219

[B22] Li Y. Peng L. Zhou Y. You Y. Jiang J. Luo Y. (2025). KAT5 knockdown alleviates microglial inflammatory injury following acute cerebral ischemia through enhanced STAT6 activity. *Brain Res. Bull.* 229:111467. 10.1016/j.brainresbull.2025.111467 40675266

[B23] Li Z. Rasmussen L. (2020). TIP60 in aging and neurodegeneration. *Ageing Res. Rev.* 64:101195. 10.1016/j.arr.2020.101195 33091598

[B24] Loosse C. Pawlas M. Bukhari H. Maghnouj A. Hahn S. Marcus K. et al. (2016). Nuclear spheres modulate the expression of BEST1 and GADD45G. *Cell. Signal.* 28 100–109. 10.1016/j.cellsig.2015.10.019 26521045

[B25] Lorbeck M. Pirooznia K. Sarthi J. Zhu X. Elefant F. (2011). Microarray analysis uncovers a role for Tip60 in nervous system function and general metabolism. *PLoS One* 6:e18412. 10.1371/journal.pone.0018412 21494552 PMC3073973

[B26] Mai H. Thomas C. Nge G. Elefant F. (2025). Modulating cognition-linked histone acetyltransferases (HATs) as a therapeutic strategy for neurodegenerative diseases: Recent advances and future trends. *Cells* 14:873. 10.3390/cells14120873 40558500 PMC12190429

[B27] Matilla-Dueñas A. Ashizawa T. Brice A. Magri S. McFarland K. Pandolfo M. et al. (2014). Consensus paper: pathological mechanisms underlying neurodegeneration in spinocerebellar ataxias. *Cerebellum* 13 269–302. 10.1007/s12311-013-0539-y 24307138 PMC3943639

[B28] Mihalas A. Arora S. O’Connor S. Feldman H. Cucinotta C. Mitchell K. et al. (2025). KAT5 regulates neurodevelopmental states associated with G0-like populations in glioblastoma. *Nat. Commun.* 16:4327. 10.1038/s41467-025-59503-w 40346033 PMC12064679

[B29] Mihaylova M. Shaw R. (2013). Metabolic reprogramming by class I and II histone deacetylases. *Trends Endocrinol. Metab.* 24 48–57. 10.1016/j.tem.2012.09.003 23062770 PMC3532556

[B30] Mousavi N. Yang X. (2025). Lysine acetyltransferase 6 complexes in neurodevelopmental disorders and different types of cancer. *Results Probl. Cell. Differ.* 75 391–410. 10.1007/978-3-031-91459-1_14 40593218

[B31] Nesbit C. Tersak J. Prochownik E. V. (1999). MYC oncogenes and human neoplastic disease. *Oncogene* 18 3004–3016. 10.1038/sj.onc.1202746 10378696

[B32] Osorio R. Oh J. Choudhary N. Lad M. Savastano L. Aghi M. (2022). Pituitary adenomas and cerebrovascular disease: A review on pathophysiology, prevalence, and treatment. *Front. Endocrinol.* 13:1064216. 10.3389/fendo.2022.1064216 36578965 PMC9791098

[B33] Panikker P. Xu S. Zhang H. Sarthi J. Beaver M. Sheth A. et al. (2018). Restoring Tip60 HAT/HDAC2 balance in the neurodegenerative brain relieves epigenetic transcriptional repression and reinstates cognition. *J. Neurosci.* 38 4569–4583. 10.1523/JNEUROSCI.2840-17.2018 29654189 PMC5943982

[B34] Penney J. Tsai L. (2014). Histone deacetylases in memory and cognition. *Sci. Signal* 7:re12. 10.1126/scisignal.aaa0069 25492968

[B35] Przedborski S. Vila M. Jackson-Lewis V. (2003). Neurodegeneration: What is it and where are we? *J. Clin. Invest.* 111 3–10. 10.1172/JCI17522 12511579 PMC151843

[B36] Sapountzi V. Logan I. Robson C. (2006). Cellular functions of TIP60. *Int. J. Biochem. Cell. Biol.* 38 1496–1509. 10.1016/j.biocel.2006.03.003 16698308

[B37] Serra H. Duvick L. Zu T. Carlson K. Stevens S. Jorgensen N. et al. (2006). RORalpha-mediated Purkinje cell development determines disease severity in adult SCA1 mice. *Cell* 127 697–708. 10.1016/j.cell.2006.09.036 17110330

[B38] Sharma R. Grayson D. Gavin D. (2008). Histone deactylase 1 expression is increased in the prefrontal cortex of schizophrenia subjects: analysis of the National Brain Databank microarray collection. *Schizophr. Res.* 98 111–117. 10.1016/j.schres.2007.09.020 17961987 PMC2254186

[B39] Shi H. Fang Y. Huang L. Gao L. Lenahan C. Okada T. et al. (2021). Activation of galanin receptor 1 with M617 attenuates neuronal apoptosis via ERK/GSK-3β/TIP60 pathway after subarachnoid hemorrhage in rats. *Neurotherapeutics* 18 1905–1921. 10.1007/s13311-021-01066-x 34086200 PMC8609084

[B40] Shi W. Cassmann T. Bhagwate A. Hitosugi T. Ip W. (2024). Lactic acid induces transcriptional repression of macrophage inflammatory response via histone acetylation. *Cell. Rep.* 43:113746. 10.1016/j.celrep.2024.113746 38329873 PMC10957222

[B41] Simon G. Moise N. Mohr D. (2024). Management of depression in adults: A review. *JAMA* 332 141–152. 10.1001/jama.2024.5756 38856993

[B42] Song Z. Lin H. Yi X. Guo W. Hu M. Shu H. (2020). KAT5 acetylates cGAS to promote innate immune response to DNA virus. *Proc. Natl. Acad. Sci. U S A.* 117 21568–21575. 10.1073/pnas.1922330117 32817552 PMC7474609

[B43] Soria Lopez J. González H. Léger G. (2019). Alzheimer’s disease. *Handb. Clin. Neurol.* 167 231–255. 10.1016/B978-0-12-804766-8.00013-3 31753135

[B44] Tarsounas M. Sung P. (2020). The antitumorigenic roles of BRCA1-BARD1 in DNA repair and replication. *Nat. Rev. Mol. Cell. Biol.* 21 284–299. 10.1038/s41580-020-0218-z 32094664 PMC7204409

[B45] Tejwani L. Lim J. (2020). Pathogenic mechanisms underlying spinocerebellar ataxia type 1. *Cell. Mol. Life Sci.* 77 4015–4029. 10.1007/s00018-020-03520-z 32306062 PMC7541529

[B46] Tirpe A. Streianu C. Tirpe S. Kocijancic A. Pirlog R. Pirlog B. et al. (2023). The glioblastoma CircularRNAome. *Int. J. Mol. Sci.* 24:14545. 10.3390/ijms241914545 37833993 PMC10572686

[B47] Tominaga K. Sakashita E. Kasashima K. Kuroiwa K. Nagao Y. Iwamori N. et al. (2023). Tip60/KAT5 histone acetyltransferase is required for maintenance and neurogenesis of embryonic neural stem cells. *Int. J. Mol. Sci.* 24:2113. 10.3390/ijms24032113 36768434 PMC9916716

[B48] Wang B. Chen D. Jiang R. Ntim M. Lu J. Xia M. et al. (2022). TIP60 buffers acute stress response and depressive behaviour by controlling PPARγ-mediated transcription. *Brain Behav. Immun.* 101 410–422. 10.1016/j.bbi.2022.01.022 35114329

[B49] Wey H. Gilbert T. Zürcher N. She A. Bhanot A. Taillon B. et al. (2016). Insights into neuroepigenetics through human histone deacetylase PET imaging. *Sci. Transl. Med.* 8:351ra106. 10.1126/scitranslmed.aaf7551 27510902 PMC5784409

[B50] Xu L. Chen Y. Song Q. Xu D. Wang Y. Ma D. (2009). PDCD5 interacts with Tip60 and functions as a cooperator in acetyltransferase activity and DNA damage-induced apoptosis. *Neoplasia* 11 345–354. 10.1593/neo.81524 19308289 PMC2657881

[B51] Xu S. Wilf R. Menon T. Panikker P. Sarthi J. Elefant F. (2014). Epigenetic control of learning and memory in Drosophila by Tip60 HAT action. *Genetics* 198 1571–1586. 10.1534/genetics.114.171660 25326235 PMC4256772

[B52] Yiliyaer N. Li X. Guo T. Zhou H. Gong L. Yuan L. et al. (2025). Pathogenicity of mediator complex subunit 27 (MED27) in a neurodevelopmental disorder with cerebellar atrophy. *Adv. Sci.* 12:e05535. 10.1002/advs.202505535 41017421 PMC12752580

[B53] You L. Zou J. Zhao H. Bertos N. Park M. Wang E. et al. (2015). Deficiency of the chromatin regulator BRPF1 causes abnormal brain development. *J. Biol. Chem.* 290 7114–7129. 10.1074/jbc.M114.635250 25568313 PMC4358132

[B54] Zhao L. Qiu Z. Yang Z. Xu L. Pearce T. Wu Q. et al. (2024). Lymphatic endothelial-like cells promote glioblastoma stem cell growth through cytokine-driven cholesterol metabolism. *Nat. Cancer* 5 147–166. 10.1038/s43018-023-00658-0 38172338

